# Deletion Variants of Middle East Respiratory Syndrome Coronavirus from Humans, Jordan, 2015 

**DOI:** 10.3201/eid2204.152065

**Published:** 2016-04

**Authors:** Mart M. Lamers, V. Stalin Raj, Mah’d Shafei, Sami Sheikh Ali, Sultan M. Abdallh, Mahmoud Gazo, Samer Nofal, Xiaoyan Lu, Dean D. Erdman, Marion P. Koopmans, Mohammad Abdallat, Aktham Haddadin, Bart L. Haagmans

**Affiliations:** Erasmus University Medical Center, Rotterdam, the Netherlands (M.M. Lamers, V.S. Raj, M.P. Koopmans, B.L. Haagmans);; Jordan Ministry of Health, Amman, Jordan (M. Shafei, S.S. Ali, S.M. Abdallh, M. Gazo, S. Nofal, M. Abdallat, A. Haddadin);; Centers for Disease Control and Prevention, Atlanta, Georgia, USA (X. Lu, D.D. Erdman)

**Keywords:** open reading frame, ORF4a, ORF3, deletion variant, Jordan, MERS-CoV, Middle East respiratory syndrome coronavirus, viruses, respiratory infections, coronaviruses

## Abstract

We characterized Middle East respiratory syndrome coronaviruses from a hospital outbreak in Jordan in 2015. The viruses from Jordan were highly similar to isolates from Riyadh, Saudi Arabia, except for deletions in open reading frames 4a and 3. Transmissibility and pathogenicity of this strain remains to be determined.

Middle East respiratory syndrome coronavirus (MERS-CoV) was first recognized in 2012 as the cause of severe lower respiratory tract infection in humans (*1*). As of November 13, 2015, a total of 1,618 cases of laboratory-confirmed MERS-CoV infection and 579 associated deaths had been reported to the World Health Organization (*2*). Human-to-human transmission of MERS-CoV can occur in households and hospitals, but thus far, major genomic changes associated with host switching, as have occurred with severe acute respiratory syndrome (SARS) CoV, have not been noted for MERS-CoV (*3*–*9*). We characterized MERS-CoVs from a recent hospital outbreak in Jordan. 

## The Study

During August and September 2015, an outbreak of MERS-CoV infection occurred in hospitals in Jordan (*10*). MERS-CoV–positive throat swab or bronchiolar lavage samples were obtained from each patient; of these, 13 samples were labeled Jordan-1-2015 through Jordan-13-2015 and shipped on dry ice to Erasmus University Medical Center (Rotterdam, the Netherlands) for testing. Total RNA was isolated from 140 μL of each sample by use of a QIAamp Viral RNA Mini Kit (QIAGEN, Hilden, Germany) and quantified by TaqMan assay targeting the envelope gene, as described previously (*11*). Full MERS-CoV genome sequencing was performed on the sample with the highest viral load, obtained on August 24, 2015, from a 60-year-old man who died of the disease 3 days later. At the end of July 2015, this man had traveled from Jeddah, Saudi Arabia, to Jordan for his annual vacation. From the throat swab sample from this man, 76,082 sequence reads—of which 851 were specific for MERS-CoV—were obtained by using 454 deep-sequencing (*12*), revealing ≈85% of the MERS-CoV genome with coverage of 1–178 reads at single-nucleotide positions. Missing sequences and low-coverage regions were obtained by conventional Sanger sequencing, except for 6 nt at the 5′ end.

**Figure 1:**
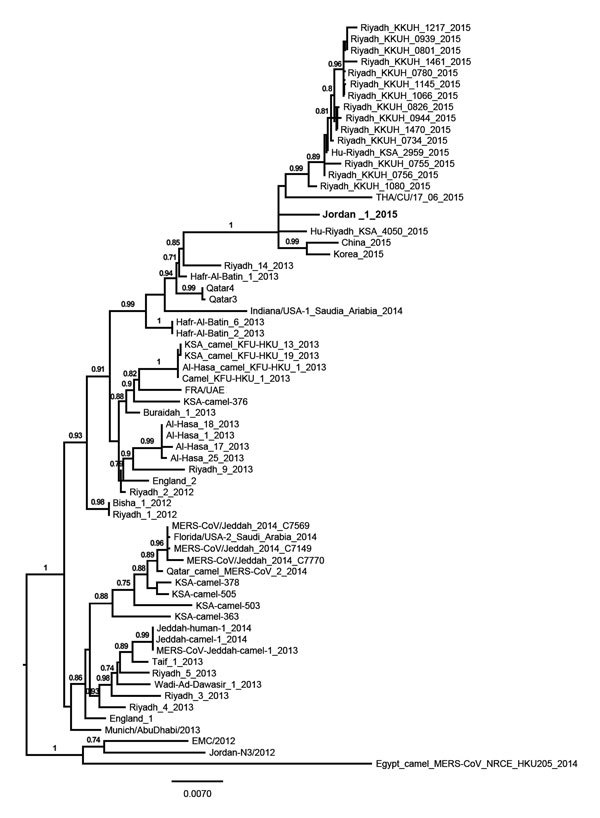
Phylogenetic analysis of Middle East respiratory syndrome coronavirus (MERS-CoV) isolated from Jordan (Jordan-1-2015; boldface) compared with reference strains. Genome sequences of representative isolates were aligned by using ClustalW, and a phylogenetic tree was constructed by using the PhyML method in Seaview 4 (http://pbil.univ-lyon1.fr/software/seaview); the tree was visualized by using FigTree version 1.3.1 (http://tree.bio.ed.ac.uk/software/figtree). Values at branches show the result of the approximate likelihood ratio; values <0.70 are not shown. Scale bar indicates nucleotide substitutions per site.

**Figure 2:**
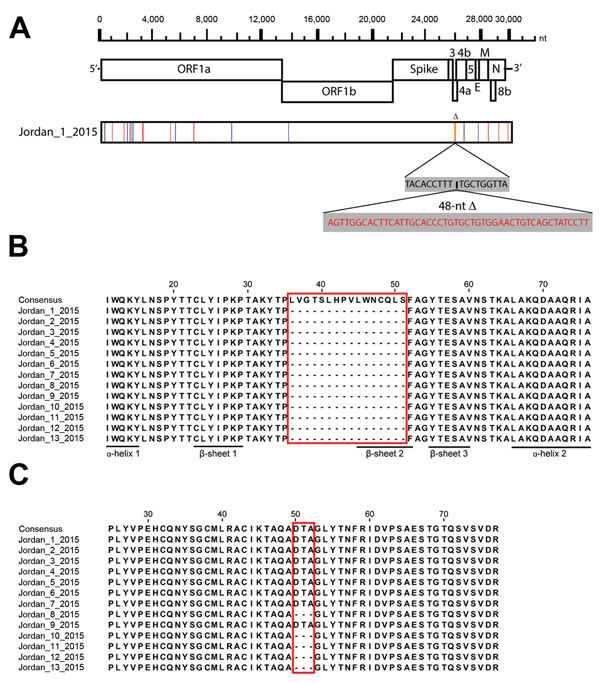
Genomic characterization of Middle East respiratory syndrome coronaviruses (MERS-CoVs) from Jordan. A) Nucleotide differences between MERS-CoV strain Jordan-1-2015 and the consensus sequence of the Riyadh 2015 cluster. Nucleotide changes are indicated in green (A), red (T), blue (C), and black (G). Deletions are indicated in orange. Deleted nucleotides are indicated in red. B) Protein sequence alignment of open reading frame (ORF) 4a (residues 10–76) of all 13 MERS-CoV strains from Jordan in 2015 compared with the consensus sequence of the Riyadh 2015 cluster. No nucleotide substitutions were observed in this region between MERS-CoVs from Jordan. Predicted secondary structures are indicated (*13*). Classical double-stranded RNA binding proteins have αβββα architecture. C) Protein sequence alignment of ORF3 (residues 25–77) of all 13 MERS-CoV strains from Jordan in 2015 compared with the consensus sequence of the Riyadh 2015 cluster. No nucleotide substitutions were observed in this region between MERS-CoVs from Jordan. Alignments were generated with the ClustalW program (http://pbil.univ-lyon1.fr/software/seaview) and visualized by using Jalview 2.9 (http://www.jalview.org/). Boxes in panels B and C indicate regions where amino acids have been deleted in the viruses from Jordan.

Phylogenetic analysis of the complete viral genome of this sample revealed that this virus, tentatively called Jordan-1-2015, clusters with viruses detected in humans in Riyadh in 2015 (Figure 1). Alignment of the full genomic sequences of this virus with the Riyadh 2015 isolates showed that they were >99.7% similar. Amino acids in the spike protein were the same in Jordan-1-2015 and the consensus sequence of the Riyadh 2015 viruses. Of note, Jordan-1-2015 contained a 48-nt (16 aa) in-frame deletion in open reading frame (ORF) 4a that had not been detected in any other MERS-CoV (Figure 2, panel A). This ORF is present only in lineage 2c betacoronaviruses and encodes protein 4a, which has recently been shown to inhibit type I interferon production, presumably by binding and masking double-stranded RNA from detection by RNA sensors such as RIG-1 (retinoic acid–inducible gene 1)–like helicases and the double-stranded RNA-binding protein PACT (protein activator of the interferon-induced protein kinase) (*13*,*14*). Subsequent sequence analysis showed that all 13 viruses from patients sampled during this outbreak contained the deletion and that the flanking regions were identical, indicating that this deletion mutant was transmitted (Figure 2, panel B).

To further characterize the viruses in the clinical samples, we subjected all samples to titration on Vero cells (American Type Culture Collection no. CCL-81). We observed cytopathic changes in cells inoculated with samples Jordan-1-2015 and Jordan-10-2015 (obtained on September 17 from a 29-year-old man, who survived the illness); however, no cytopathic changes were observed in the cells inoculated with the other samples. Subsequently, we obtained complete genomes by deep sequencing of the second-passage viruses, with high coverage of reads at single-nucleotide positions. No cell culture adaptive mutations in the genome sequence were observed for the virus isolated from clinical sample Jordan-1-2015. Pairwise comparison of Jordan-1-2015 with Jordan-10-2015 revealed that both viruses were highly similar (99.99% nt identity) and confirmed that both carried the 48-nt deletion in ORF4a. However, in addition to this deletion, a 9-nt (3-aa) in-frame deletion was observed in the genome of Jordan-10-2015. This deletion was located in ORF3, which encodes a protein with an unknown function. The presence of this deletion in the virus from clinical sample Jordan-10-2015 was confirmed by Sanger sequencing, excluding the possibility that it was an adaptation to cell culture. Next, sequencing of ORF3 from all other clinical samples revealed that the ORF3 deletion was present in a subgroup of clinical samples (Figure 2, panel C). These samples had been collected toward the end of the outbreak, suggesting that the ORF3 deletion may be an adaptation resulting from sustained transmission between humans. All sequences obtained in this study were deposited in GenBank (accession nos. KU233362–KU233377). Identical deletions in ORF3 and ORF4a in the same sample set from Jordan were independently confirmed by Erasmus University Medical Center and the Centers for Disease Control and Prevention (Atlanta, GA, USA).

## Conclusions

Despite the human-to-human transmission capacity of MERS-CoV and substantial sequencing efforts, major genomic changes associated with human adaptation have not been described (*3*–*8*). We observed a large-scale deletion in ORF4a and a small deletion in ORF3 in MERS-CoVs that caused the outbreak in Jordan. 

One of the most striking genomic changes observed in SARS-CoV isolated from humans soon after its transmission from animals was the acquisition of a 29-nt deletion in ORF8, an accessory protein with an unresolved function (*9*). The SARS-CoV ORF8 deletion disrupted the reading frame of the early-stage human isolates and created 2 new ORFs, designated ORF8a and ORF8b. Although no clear roles have been designated for both ORFs, viruses containing this deletion predominated later during the epidemic, showing that these viruses were able to spread efficiently from human to human. 

The deletion we describe in ORF4a of MERS-CoV does not result in the removal of an entire gene, because the reading frame is not disrupted, suggesting that this mutation is not a loss-of-function mutation. However, whether this ORF4a deletion variant can still bind double-stranded RNA needs to be assessed because the deleted region contains a predicted β-sheet belonging to the classical double-stranded RNA binding αβββα fold of this protein (*13**;* Figure 2, panel B). The data from this study indicate that adaptive pressures possibly exerted by the human host may operate on the ORF4a and ORF3 regions. Alternatively, proteins encoded by these ORFs could have functions specific for the camel host, causing them to be redundant in humans and enabling accumulation of mutations that do not affect viral fitness in humans. Future studies of the roles of these proteins are needed.

The deletion located in the type I interferon antagonist protein 4a was detected in all samples collected during the outbreak. Another small-scale deletion was detected in ORF3 in a subset of samples collected later in the outbreak. Overall, the data suggest that MERS-CoV ORF4a and ORF3 can acquire deletions by selection or by chance. Whether these deletions affect the transmissibility or pathogenicity of this particular MERS-CoV strain needs to be addressed. The finding that all viruses analyzed contained the ORF4a deletion suggests that all patients were infected with the same virus. Although unlikely, some MERS patients may have indirectly become infected through independent transmission of viruses in dromedaries carrying highly related viruses, some of which acquired deletions in their genome. Therefore, a more detailed epidemiologic investigation of this outbreak is needed. These data underscore the need for close monitoring of the molecular evolution of MERS-CoV.
